# Perioperative Cannabinoids Significantly Reduce Postoperative Opioid Requirements in Patients Undergoing Coronary Artery Bypass Graft Surgery

**DOI:** 10.7759/cureus.58566

**Published:** 2024-04-18

**Authors:** Ujjawal Kumar, Antoni R Macko, Nayoung Kang, Nicole G Darian, Ferena O Salek, Zain Khalpey

**Affiliations:** 1 Clinical Medicine, University of Cambridge, Cambridge, GBR; 2 Cardiothoracic Surgery, HonorHealth, Scottsdale, USA; 3 Surgery, Midwestern University Arizona College of Osteopathic Medicine, Glendale, USA; 4 Pharmacy, Providence St. Joseph Hospital Orange, Orange, USA; 5 Pharmacy, Northwest Medical Center, Tucson, USA

**Keywords:** perioperative pain management, synthetic cannabinoids, coronary artery bypass graft surgery, analgesia adjunct, postoperative pain relief

## Abstract

Background

Opioids, commonly used to control pain associated with surgery, are known to prolong the duration of mechanical ventilation and length of hospital stay. A wide range of adjunctive strategies are currently utilized to reduce postoperative pain, such as local and regional nerve blocks, nerve cryoablation, and adjunctive medications. We hypothesized that dronabinol (a synthetic cannabinoid) in conjunction with standard opioid pain management will reduce opioid requirements to manage postoperative pain.

Methods

Sixty-eight patients who underwent isolated first-time coronary artery bypass graft surgery were randomized to either the control group, who received only standard opioid-based analgesia, or the dronabinol group, who received dronabinol (a synthetic cannabinoid) in addition to standard opioid-based analgesia. Dronabinol was given in the preoperative unit, before extubation in the ICU, and after extubation on the first postoperative day. Preoperative, intraoperative, and postoperative parameters were compared under an IRB-approved protocol. The primary endpoints were the postoperative opioid requirement, duration of mechanical ventilation, and ICU length of stay, and the secondary endpoints were the duration of inotropic support needed, left ventricular ejection fraction (LVEF), and the change in LVEF. This study was undertaken at Northwest Medical Center, Tucson, AZ, USA.

Results

Sixty-eight patients were randomized to either the control group (n = 37) or the dronabinol group (n = 31). Groups were similar in terms of demographic features and comorbidities. The total postoperative opioid requirement was significantly lower in the dronabinol group [39.62 vs 23.68 morphine milligram equivalents (MMEs), p = 0.0037], representing a 40% reduction. Duration of mechanical ventilation (7.03 vs 6.03h, p = 0.5004), ICU length of stay (71.43 vs 63.77h, p = 0.4227), and inotropic support requirement (0.6757 vs 0.6129 days, p = 0.7333) were similar in the control and the dronabinol groups. However, there was a trend towards lower durations in each endpoint in the dronabinol group. Interestingly, a significantly better preoperative to postoperative LVEF change was observed in the dronabinol group (3.51% vs 6.45%, p = 0.0451).

Conclusions

Our study found a 40% reduction in opioid use and a significantly greater improvement in LVEF in patients treated with adjunctive dronabinol. Mechanical ventilation duration, ICU length of stay, and inotropic support requirement tended to be lower in the dronabinol group, though did not reach statistical significance. The results of this study, although limited by sample size, are very encouraging and validate our ongoing investigation.

## Introduction

Approximately two million cardiac surgical procedures via a full median sternotomy approach are performed globally each year; these are associated with significant postoperative pain [1,2]. Opioids play a central role in cardiac perioperative care and continue to be a primary modality for postoperative analgesia in cardiac surgical patients [3]. The management of postoperative pain is a complex and challenging endeavor that requires careful administration and continuous vigilant monitoring to optimize analgesic effects and patient comfort while minimizing adverse and potentially harmful consequences.

On one hand, inadequate analgesia and poorly controlled pain following median sternotomy are associated with sympathetic nervous system activation, increased endocrine stress response, and numerous adverse postoperative outcomes including decreased mobilization, increased respiratory complications, myocardial ischemia, cardiac arrhythmias, delirium, and prolongation of hospital stay [4,5]. On the other hand, escalating doses of opioids are known to cause cardiovascular, respiratory, and gastrointestinal complications. They may also lead to extended duration of mechanical ventilation and hospital stay. Paradoxically, the use of high doses of opioids can also worsen pain via opioid-induced hyperalgesia [6,7]. Postoperative ileus, which occurs in about 10% of cardiac surgeries, also results in increased length of stay, increased healthcare costs, and higher readmission rates [8]. Additionally, opioid use exposes the patient to the potential risk for the development of dependence and future abuse [3,9].

While numerous opioid analgesics have been developed, thoroughly studied, and have become a fundamental component of the institutional standard of care due to their powerful analgesic effects, relatively predictable pharmacokinetics, cost, availability, and generally acceptable side effect profiles, it is the opinion of our group and others, as evidenced in the literature, that alternate strategies to better mitigate pain while decreasing opioid use warrant investigation [3,4]. In addition to non-steroidal anti-inflammatory drugs (NSAIDs), some of the proposed strategies to mitigate pain following median sternotomy include prolonged or continuous local and regional parasternal nerve blocks, cryoablation of intercostal nerves, and the use of pharmacological adjuncts such as cannabinoids, as investigated in this study [4,10].

Given what is currently known about cannabinoids regarding pharmacologic properties, mechanisms of action, synergistic effects with opioids, and generally good tolerance reported in previously published studies, we sought to elucidate the potential role for perioperative administration of a synthetic cannabinoid as an analgesic adjunct for cardiac surgery patients. Dronabinol is a synthetic derivative of delta-9-tetrahydrocannabinol (delta-9-THC) and binds to the CB1 and CB2 cannabinoid receptors. There are no published reports in the literature on the use of cannabinoids as a perioperative analgesic adjunct with traditional opioid-based therapy in the context of open-heart surgery with median sternotomy.

We hypothesize that dronabinol, in conjunction with opioid pain management, will reduce the total opioid requirement to manage postoperative pain, mechanical ventilation duration, and the duration of admission to the intensive care unit (ICU) in patients undergoing open heart surgery. Findings from this study were previously presented in part at the Society of Thoracic Surgeons Coronary Conference on the 3rd and 4th of June 2023 and the Society of Cardiothoracic Surgery in Great Britain and Ireland’s Annual Conference on the 18th of March 2024.

## Materials and methods

This is a prospective randomized cohort study, comprised of patients who underwent isolated first-time coronary artery bypass graft (CABG) surgery via median sternotomy at Northwest Medical Center, Tucson, AZ, USA. Institutional Review Board ethical approval was granted for outcome analysis in this study (IRB#20200195/02/05/2020) and informed consent was obtained for all patients for the relevant surgical procedures and anonymized inclusion into this study. All methods of this study were conducted as per the relevant guidelines and regulations for working with human subjects. All patients included were over the age of 18. Exclusion criteria included pre-existing diagnoses of heart failure and chronic kidney disease. Patients with previous long-term cannabis or opioid use or any contraindications for cannabinoid therapy were also excluded.

Heart failure was defined using American Heart Association (AHA) criteria for diastolic heart failure as a left ventricular ejection fraction (LVEF) less than or equal to 40% by transthoracic echocardiography [11]. Chronic kidney disease (CKD) was defined using an internationally conventional set of guidelines from Kidney Disease: Improving Global Outcomes (KDIGO). These criteria define chronic kidney disease as having a glomerular filtration rate of less than 60 ml/min/1.73m^2^ and albuminuria with an albumin-creatinine ratio greater than 3 mg/mmol [12]. Contraindications for dronabinol therapy were considered as per the Food and Drug Administration (FDA) guidance [13].

Data collection

Demographic data, preoperative clinical characteristics, operative characteristics, and postoperative outcomes were collected from the institutional electronic health record system (Epic). Data collected was anonymized and stored on a secure server as per institutional information governance protocols for outcomes research data. Pre-operative characteristics collected also included comorbidities such as hypertension (HTN), chronic obstructive pulmonary disease (COPD), prior myocardial infarction (MI), cerebrovascular accident/stroke (CVA), or sternotomy, as well as key risk scores such as CHA_2_DS_2_-VASc and HAS-BLED.

Postoperatively, data was collected for the total opioid usage during hospital admission; as the exact regimen of opioid analgesia varied between patients, this was standardized for comparison by conversion into morphine milligrams equivalent (MME) using the Centers for Disease Control and Prevention (CDC) opioid conversion factors [14]. Primary endpoints analyzed included morphine milligram equivalents (MME) utilized to achieve adequate pain control postoperatively, duration of mechanical ventilation, and ICU length of stay (LOS).

Additionally, secondary endpoints investigated were the number of days of inotropic support required, as well as a group comparison of preoperative and postoperative LVEF, and more importantly the change between preoperative and postoperative LVEF. Preoperative LVEF was measured in the operating room using transesophageal echocardiography before the surgery started and postoperative LVEF was measured at hospital discharge. Inotropic support was provided using either dobutamine or low-dose norepinephrine (Levophed), as is standard at our institution. The secondary endpoints were defined at the time of study design and were of interest as there are published reports in the literature detailing the reduction of cardiac function with opioid therapy. At the analysis stage, any patients who had received any form of mechanical circulatory support (intra-aortic balloon pump or ventricular assist device) were retrospectively excluded from the study; these patients may have significantly longer ICU admissions, potentially confounding this key endpoint in our study.

Operative technique and clinical protocol

**Figure 1:**
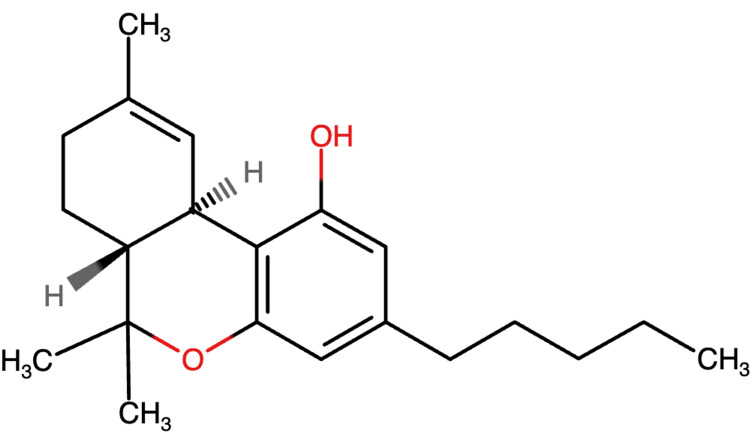
Molecular structure of dronabinol, the synthetic cannabinoid investigated in this study. This figure was created using the Research Collaboratory for Structural Bioinformatics Protein Data Bank (RCSB PDB) chemical sketch tool, using the chemical and structural formula [15].

All patients included in this study were undergoing isolated first-time coronary artery bypass graft surgery. All patients gave full informed consent for enrollment in this study and were adequately informed of the risks and benefits of the procedures as well as the respective postoperative analgesia protocols. Patients were randomized into two groups: the control group (n = 37), who received only conventional opiate-based analgesia postoperatively, and the dronabinol group (n = 31), who received a total dose of 90 mg of dronabinol (Marinol™, Unimed Pharmaceuticals, High Point, NC, USA, molecular structure in Figure 1) in three single doses of 30 mg each.

The dronabinol group received their first dose of dronabinol in the preoperative unit, the second dose before extubation in the ICU, and the third and final dose after extubation on postoperative day one. Postoperative analgesia was prescribed following local guidelines adhering to the WHO analgesic ladder [16]. First, patients were prescribed the maximal dose of acetaminophen (1 g four times daily), with or without the appropriate use of nonsteroidal analgesia, then progressing to weak, and then stronger opioids. All patients, regardless of group, were provided standard opioid-based postoperative analgesia in the form of morphine, hydromorphone, oxycodone, tramadol, and fentanyl either in isolation or in various combinations to adequately control pain and as deemed appropriate as per unique patient considerations and tolerances. No patients were discharged home from the cardiac surgical department with any opioid medications.

Statistical analysis

Data were summarized using descriptive statistics. For continuous variables, the mean and standard error in the mean (SEM) were presented for parametric data while the median and interquartile range (IQR) were presented for non-parametric data. Categorical variables were presented as N (%). Group differences were investigated using t-tests (parametric) or Mann-Whitney U test (non-parametric) for continuous variables. Categorial variables were compared using Chi-Square or Fisher exact (for frequency cells < 5) tests. All statistical analyses were performed using GraphPad Prism version 10.1.0 for macOS (GraphPad Software, Boston, MA, USA), and a p-value of 0.05 was considered the significance threshold, as is conventional [17]. All p-values displayed are calculated by comparing the dronabinol group to the control group.

## Results

**Figure 2:**
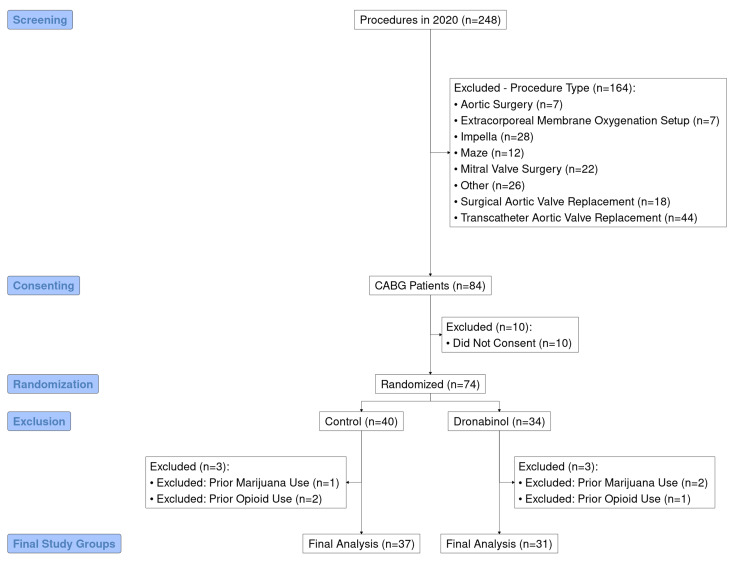
CONSORT flow diagram highlighting the selection process for this study. After strictly applying the inclusion and exclusion criteria to our study population of 248 patients, the final sample size was 68 patients. This diagram was created using the “consort” package in the R statistical software (v4.3.3) [18,19].

The patient selection and inclusion process for this study is depicted in Figure 2. First, patients undergoing any cardiac surgical procedure other than isolated first-time coronary artery bypass graft surgery were excluded (n = 164). Of the remaining 84 patients, 10 did not consent to inclusion in this study, leaving 74 who were randomized. Three patients in each of the two study groups were excluded as per our defined exclusion criteria of prior long-term opioid or marijuana use. After strictly applying these inclusion and exclusion criteria, the final sample size was therefore 68. Thirty-one patients were randomized to the dronabinol group, with the remaining 37 patients randomized to the control group.

**Figure 3:**
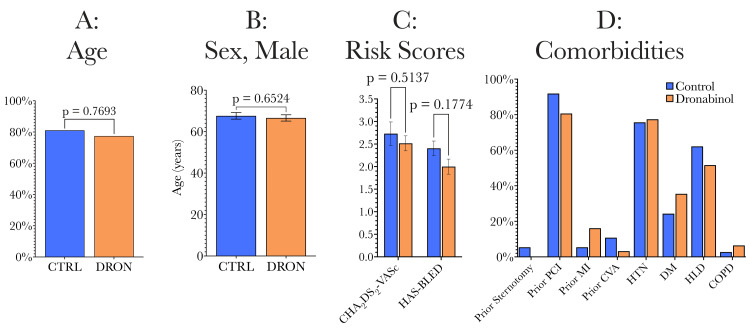
Comparison of preoperative characteristics (demographics and comorbidities). The two study groups were similar in terms of mean age, sex ratio, key risk scores, as well as co-morbidities/past medical history. CHA_2_DS_2_-VASc: a scoring system to assess stroke risk, HAS-BLED: a scoring system to assess the risk of major bleeding for patients on anticoagulation, PCI: percutaneous coronary intervention/stenting, MI: myocardial infarction, CVA: cerebrovascular accident/stroke, HTN: hypertension, DM: diabetes mellitus, HLD: hyperlipidemia, COPD: chronic obstructive pulmonary disease.

Group comparisons of patient characteristics are summarized in Figure 3, with full comparisons shown in Table 1. When comparing the dronabinol and control groups in terms of demographic and preoperative clinical characteristics and co-morbidities, there were no significant differences. Groups were therefore well-matched in terms of age (Figure 3A), gender (Figure 3B), their CHA_2_DS_2_-VASc and HAS-BLED risk scores (Figure 3C), as well as past medical history/comorbidities (Figure 3D).

**Table 1:** Demographics and baseline characteristics for patients included in this study. No significant differences between the dronabinol and control groups in any demographic or clinical characteristics. LVEF: left ventricular ejection fraction, BMI: body mass index, CHA_2_DS_2_-VASc: a scoring system to assess stroke risk, HAS-BLED: a scoring system to assess the risk of major bleeding for patients on anticoagulation, PCI: percutaneous coronary intervention/stenting, MI: myocardial infarction, CVA: cerebrovascular accident/stroke, HTN: hypertension, DM: diabetes mellitus, HLD: hyperlipidemia, COPD: chronic obstructive pulmonary disease.

Variable	Control Group	SEM / IQR	%	Dronabinol Group	SEM / IQR	%	p-value
Number	37			31			N/A
Demographics							
Age (years)	67.62	9.66		66.61	8.52		0.6524
Sex: Male	30		81.08	24		77.42	0.7693
Comorbidities							
Pre-op LVEF (%)	55	45-60		50	45-55		0.3360
BMI (kg/m^2^)	28.00	25.00-30.00		28.00	25.00-31.00		0.5471
CHA_2_DS_2_-VASc	3	2-4		3	2-3		0.5087
HAS-BLED	2	2-3		2	1-3		0.0878
Prior Sternotomy	2		5.41	0		0.00	0.4965
Prior PCI	34		91.89	25		80.65	0.2819
Prior MI	2		5.41	5		16.13	0.2325
HTN	28		75.68	24		77.42	> 0.9999
DM	9		24.32	11		35.48	0.4240
Prior CVA	4		10.81	1		3.23	0.3662
HLD	23		62.16	16		51.61	0.4361
COPD	1		2.70	2		6.45	0.5880

**Figure 4:**
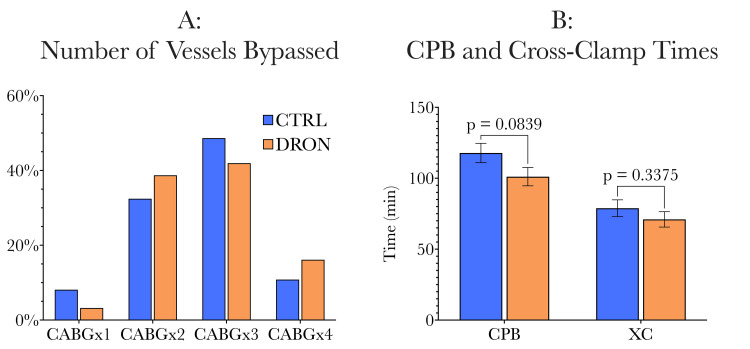
Comparison of operative characteristics (number of vessels bypassed, total cardiopulmonary bypass, and aortic cross-clamp times). The two study groups were similar in terms of the number of vessels bypassed, the cardiopulmonary bypass time, and the aortic cross-clamp time. CABG: coronary artery bypass graft, CPB: cardiopulmonary bypass, XC: aortic cross-clamp.

The control and dronabinol groups were similar when operative characteristics were compared; similar numbers of vessels were bypassed (Figure 4A), with both groups having similar cardiopulmonary bypass times and aortic cross-clamp times (Figure 4B). A comparison of operative characteristics is shown in Figure 4, with a full comparison in Table 2.

**Table 2:** Operative characteristics by group. Operative characteristics were similar between the dronabinol and the control group.

Variable	Control Group	% / SEM	Dronabinol Group	% / SEM	p-value
Number of vessels bypassed					0.7382
1	3	8.11	1	3.23	
2	12	32.43	12	38.71	
3	18	48.65	13	41.94	
4	4	10.81	5	16.13	
Cardiopulmonary bypass time, min	117.80	6.81	101.10	6.45	0.0839
Aortic cross-clamp time, min	78.86	5.91	71.00	5.42	0.3375

**Figure 5:**
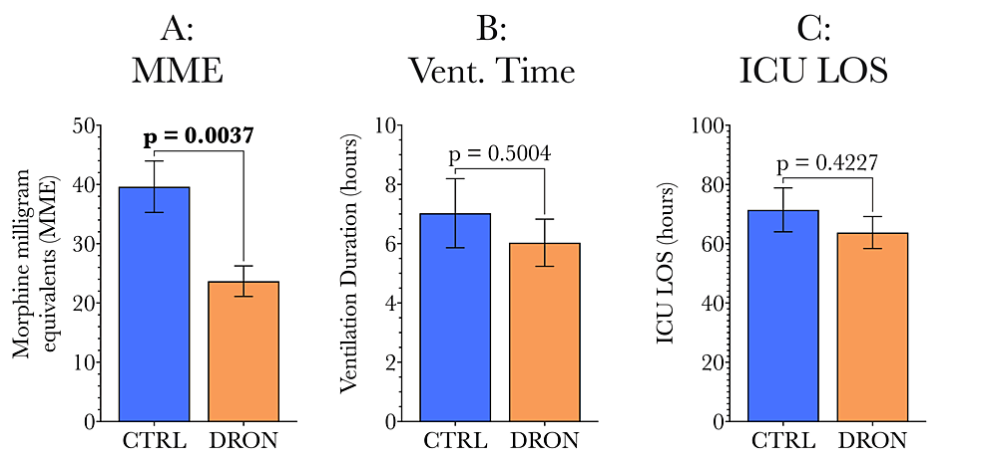
Comparison of primary endpoints (total postoperative opioid requirement, duration of mechanical ventilation, and ICU LOS) between control and dronabinol groups. The total opioid requirement was significantly lower in the dronabinol group than the control group (23.68 vs 39.62 MMEs, p = 0.0037), representing a 40% reduction in opioid usage for patients who received adjunctive dronabinol treatment. The duration of mechanical ventilation and the length of ICU admission were similar between groups. MME: morphine milligram equivalent, ICU LOS: intensive care unit length of stay.

Considering the primary endpoints shown in Figure 5, the dronabinol group showed a significant (40%) reduction in opioid requirement compared with the control group (Figure 5A). All patients tolerated dronabinol treatment well, with no adverse effects or outcomes related to dronabinol administration. The duration of mechanical ventilation (Figure 5B) and intensive care unit admission (Figure 5C) were similar between the two study groups. While these group differences did not reach statistical significance at the 5% level, it is important to note that both endpoints trended lower in the dronabinol group as can be seen in Figure 5.

**Figure 6:**
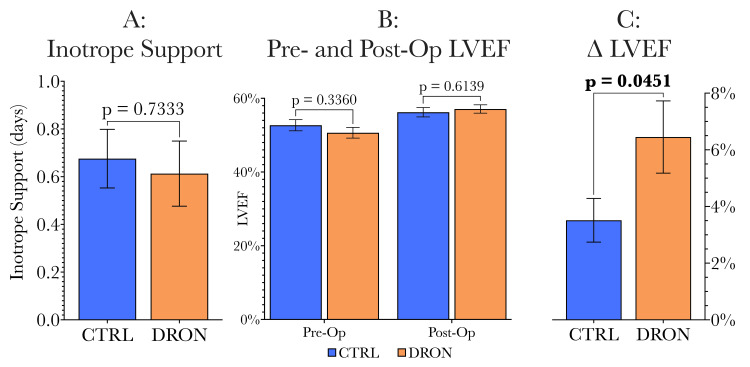
Comparison of secondary endpoints (duration of inotropic support, comparisons of preoperative and postoperative LVEF, change from preoperative to postoperative LVEF) between control and dronabinol groups. The duration of inotropic support requirement, as well as preoperative and postoperative LVEF values, were similar between the groups. However, when considering the change from preoperative LVEF to postoperative LVEF for each patient in each of the two study groups, there was a significantly greater improvement in LVEF in the dronabinol group (6.45% vs 3.51%, p = 0.0451). LVEF: left ventricular ejection fraction.

Next, secondary endpoints were compared between groups (Figure 6). The dronabinol group tended to require a shorter duration of inotropic support (Figure 6A). When comparing the groups in terms of their preoperative LVEF, as well as comparison of postoperative LVEF, the dronabinol and control groups were similar (Figure 6B). However, when considering the change for each patient from preoperative LVEF to postoperative LVEF (∆LVEF), the dronabinol group showed a significantly greater improvement in LVEF than the control group (6.45% vs 3.51%, Figure 6C).

A full comparison of primary and secondary endpoints (outcomes) is shown below in Table 3.

**Table 3:** Primary and secondary endpoints (outcomes) by group. The total opioid requirement was significantly lower in the dronabinol group than in the control group (23.68 vs 39.62 MMEs, p = 0.0037). The duration of mechanical ventilation, ICU admission, and inotropic support were similar between groups. Comparing the change from preoperative LVEF to postoperative LVEF for each patient, the dronabinol group showed a significantly greater improvement in LVEF (6.45% vs 3.51%, p = 0.0451). MME: morphine milligram equivalent, ICU LOS: intensive care unit length of stay, LVEF: left ventricular ejection fraction

Outcome	Control Group	SEM	Dronabinol Group	SEM	p-value
Primary Endpoints					
Morphine milligram equivalents	39.62	4.22	23.68	2.57	0.0037
Mechanical ventilation duration (hours)	7.03	1.17	6.03	0.80	0.5004
ICU LOS (hours)	71.43	7.41	63.77	5.40	0.4227
Secondary Endpoints					
Inotropic support (days)	0.6757	0.12	0.6129	0.14	0.7333
ΔLVEF (%)	3.51	0.77	6.45	1.27	0.0451

## Discussion

Cannabinoids were first approved by the Food and Drug Administration (FDA) in 1985 to treat chemotherapy-induced emesis in patients with various malignancies and to stimulate appetite in patients with AIDS-related anorexia with their analgesic properties garnered increasing attention in recent years [13,20]. However, despite the burgeoning interest in the analgesic properties of cannabinoids, many questions remain, and much work remains necessary to provide the needed answers, particularly in the context of highly specialized patient populations (such as acute postoperative pain following open heart surgery). Most of the current published literature focuses on chronic pain or inhaled cannabis rather than acute postoperative pain and oral cannabinoids. When examining the literature, it is important to note the timing of treatment relative to initiation of pain. For example, in one study reporting a lack of analgesic efficacy of delta-9-THC in the context of postoperative pain, the treatment was not administered until 48 hours after the procedure, at which point the investigators arguably missed the window of opportunity for the treatment to have any meaningful impact on pain relief or opioid use [21].

Our study found that perioperative administration of the synthetic cannabinoid dronabinol significantly reduced opioid requirements by 40% and led to greater improvement in postoperative cardiac function compared to opioids alone in patients undergoing coronary artery bypass grafting (CABG). These findings suggest that cannabinoids may be a useful adjunct to traditional opioid-based pain management after cardiac surgery. Our results build upon previous research suggesting that combining opioids and cannabinoids may have synergistic effects on pain relief, potentially allowing reduced opioid dosages [22-25]. The mechanisms underlying this opioid-sparing effect likely stem from interactions between the endocannabinoid and opioid systems.

Cannabinoids exert their analgesic effects primarily via CB1 receptors in the central nervous system and CB2 receptors in the peripheral nervous system [26]. Cannabinoids, via binding to their receptors, act to inhibit the release of neurotransmitters from presynaptic nerve endings, modulate postsynaptic excitability, activate descending inhibitory pain pathways, and reduce neuronal inflammation. Importantly, CB1 receptors are expressed at low levels in brainstem cardiorespiratory centers, explaining why cannabinoids do not suppress respiratory drive like opioids do when acting on mu-opioid receptors in those same regions [27-29]. The high density of CB1 receptors close to opioid receptors in nociceptive pathways of the brain and spinal cord likely mediates their synergistic analgesic effects [30].

In addition to reducing opioid requirements, dronabinol led to a greater improvement in left ventricular ejection fraction (LVEF) after CABG compared to the control group. There is currently no consensus in the literature on the effects on cardiac function of opioids, with significant published literature suggesting cardiac suppression as a result of opioid therapy, as discussed by Chen et al., who find a particular decrease in cardiac function when opioids are used with benzodiazepines and other medications used in general anesthesia [31]. The exact mechanism for this is unclear, however, preclinical studies suggest that kappa-opioid receptor stimulation decreases calcium sensitivity in the cardiomyocyte [32]. This would lead to reduced contractility and would explain the reduction in cardiac function suggested with opioids. Since our dronabinol group received a significantly lower amount of opioid medication, this cardiac suppression effect by opioids would be lower in these patients, and therefore the improvement in LVEF would be greater, as seen in our data. Larger studies using more sophisticated echocardiographic or hemodynamic assessments are needed to confirm and further characterize this cardioprotective effect, which could have important implications for postoperative recovery.

Our findings that mechanical ventilation duration and ICU LOS were similar between groups, not reduced in the dronabinol group as we had hypothesized, may be the result of a relatively small sample size. Alternatively, this may simply reflect the fact that a variety of factors independent of analgesic agents utilized determine these outcomes, with several non-pain variables also contributing. Additionally, we observed a trend towards decreased postoperative ileus in the dronabinol group based on the clinical assessments of nurses and physicians caring for the patients. However, this outcome was not pre-specified and could be subject to observer bias. Future studies should systematically evaluate ileus using validated measures. Importantly, dronabinol appeared to be well-tolerated in this patient population. While a few patients treated with dronabinol developed very mild, transient confusion, this observation was limited to two patients, both of whom were older than 75 years of age. This low incidence rate of postoperative delirium is reassuring given concerns about the cognitive effects of cannabinoids in older adults [33,34].

Our study also found an insignificant rate of arrhythmias in the dronabinol group, particularly ventricular arrhythmias such as QT interval prolongation. This is of particular importance as there is currently a degree of debate in the literature regarding whether synthetic cannabinoids confer an increased risk for cardiac arrhythmias [35]. Of course, larger trials are warranted to fully establish the safety profile of dronabinol in this setting.

While our results are encouraging, showing value in the use of dronabinol as a perioperative analgesic adjunct, there are a few limitations to our study. The single-center nature of this study as well as the relatively small sample size may limit generalizability and statistical power for some secondary endpoints. We tried to minimize bias by using a randomized design and standardized protocols. Additionally, this study focused on short-term outcomes in patients undergoing CABG, so the results may not apply to other procedures or long-term recovery. Discussion of our long-term outcomes, as well as our experience with dronabinol for patients undergoing other cardiac surgical procedures is forthcoming in future work from our group. It is important to note that these limitations are common in pilot or proof-of-concept studies and therefore should not necessarily detract from our findings. They underscore the need for larger, multicenter, studies to confirm the efficacy and safety of dronabinol in this setting.

This study provides preliminary evidence that the addition of dronabinol to a standard opioid-based pain regimen after CABG is safe and reduces opioid requirement while also potentially conferring cardioprotective effects. These results are encouraging, and therefore validate our group’s ongoing research to elucidate methods for optimizing postoperative pain control while reducing reliance on opioids. This study also emphasized the importance of approaching postoperative pain using a multidisciplinary strategy - specialist pain pharmacists and anesthesiologists who are experienced in non-opioid analgesia were crucial to this study and our ongoing clinical practice. Our findings justify larger multicenter randomized controlled trials to confirm the efficacy and safety of this approach. If validated, cannabinoids could become an important component of multimodal pain management strategies aimed at reducing opioid exposure and improving outcomes after cardiac surgery.

## Conclusions

In this randomized study of coronary artery bypass patients, perioperative dronabinol administration significantly reduced opioid requirements by 40% and led to a greater improvement in postoperative cardiac function, without any adverse effects or outcomes. While the duration of ventilation and ICU stay were unaffected, the results demonstrate dronabinol's promise as a safe, opioid-sparing analgesic adjunct. The success of this multidisciplinary effort provides a strong rationale for larger multicenter trials to further investigate the feasibility of cannabinoid-based postoperative analgesic adjuncts and ultimately improve patient outcomes after cardiac surgery.
